# Notes and Notices

**Published:** 1895-06

**Authors:** 


### NOTES AND NOTICES.

A Bureau of the Organon and Homœopathic Philosophy has been established at the last meeting of the Indiana Institute of Homœopathy. "This is the first State institute to create such a bureau," writes Dr. W. R. Bentley, of Morristown, Indiana. It shows that the Indiana Institute is resolved that the position of its members on the great question of pure Homœopathy shall not be misunderstood.

A Portrait of Bœnninghausen of undoubted genuineness has been secured after a long search by Dr. John Arshagouni, of New York, who has reproduced it as a photogravure 12¼ by 15½ inches and printed on steel-plate paper 20¼ by 24¼ inches.

The price of this fine work of art is $2.00 by registered mail to any address. Address, John Arshagouni, M. D., P. O. Box 2331, New York City.

The Physicians' Insurance Association.—It is an unfortunate fact well known to the medical profession, that a majority of practicing physicians are unable to accumulate from a lifetime of professional work, a competency, and that too often, when death removes them from this earthly sphere, they leave behind them nothing but their individual debts and worthless bills.

In order that these conditions may be bettered, and that a physician may feel that he has in a measure provided in the event of his death for those who are near and dear to him, it has been suggested that the formation of a National Mutual Insurance Association, would be practicable, composed of physicians and pharmacists of the United States, and organized upon a co-operative basis, as follows:

The organization should be incorporated under State and Territorial laws, by the name, style, and title of "THE PHYSICIANS' INSURANCE ASSOCIATION."

All practitioners of Homœopathy who are graduated physicians and pharmacists, in good professional standing, shall be elegible to membership, without regard to age, sex, color, or health. (Eclectic physicians also might be made elegible, in localities where there are few homœopathists.)

In the event that the profession in a majority of the States and Territories shall deem it expedient to form such an association, and have a death benefit fund in common, such State or Territorial Association shall each thereupon become sovereign within its own boundaries.

Such State associations shall be known as Chapters, and each Chapter shall be entitled to elect annually from among their membership, one representative to a national or supreme body, which shall be known as the Supreme Chapter, and said Supreme Chapter shall meet in annual session at the same time and place as The American Institute of Homœopathy.

APPENDICITIS AND THE GRAPE SEED.—Dr. J. D. Bryant says (*Medical Record*, New York), that he had the appendix examined in one hundred and fifty autopsies, and in not one was it found to contain grape seed or any other foreign body except inspissated fæces or muco-pus. He says also that "eminent surgeons had operated for appendicitis after weighing all points, and found that appendicitis did not exist at all."—*Texas Medical Journal.*

THE *Medical Century* has removed from 31 Washington Street, to 161 Oakwood Boulevard. Mail and express matter should be addressed accordingly.

THE CHILDREN'S HOMŒOPATHIC HOSPITAL, OF PHILADELPHIA, has just elected three new resident physicians for the ensuing year: Dr. Herman A. Newbold, chief, and Drs. H. C. Hunsicker and Frank Sraganga, associates.

Last year there were 22,997 applicants for relief at the various special clinics in the Out-Patient Department, and the resident physicians made 3,263 visits. One hundred and eighty-four children were cared for in the wards. The institution recently received a legacy of $15,000 from the late Walter Garrett, a patient of Dr. Bushrod W. James and of Dr. M. D. Youngman, while residing in Atlantic City Mr. Garrett also left a legacy of $50,000 to the Hahnemann Hospital, of Philadelphia.

THE MEDICAL EXAMINER has removed to new and commodious offices at No. 78 Reade St., New York. The Editor says: "At this address we shall ever welcome our friends who call upon us. To our out-of-town correspondents and patrons, who occasionally visit the metropolis, and to Medical Examiners in general, we tender the use of the facilities our new office affords whenever they can be made available. There are desks at your disposal, long-distance telephone, good messenger service, and a competent stenographer if you require one. We are one block from Broadway, and just above City Hall Park. Drop in on us at first opportunity; always some one to receive you."

PORTRAIT OF HAHNEMANN.—Having been enabled by chance, in Paris, to come into possession of the last copy of the portrait of the justly celebrated Dr. Samuel Hahnemann, by Maurir, the officers of The San Francisco Homœopathic Polyclinic have caused to be executed by one of the leading lithographers of the Coast, a fine and greatly enlarged tinted lithographic copy of the same, and are thus able to reproduce this admirable likeness at a minimum cost, and this Polyclinic is now prepared to furnish to the profession, by mail, properly packed in a mailing tube, on receipt of $1.00 each, this well-executed (19x24) portrait of the Master. Apply to D. Albert Hiller, M. D., 220 Montgomery Avenue, San Francisco, Cal.

AMERICAN MEDICAL PUBLISHERS.—This Association held its second annual meeting at the Eutaw House, on the 6th and 7th of May, with the following in attendance:

Dr. J. C. Culbertson, Cincinnati, Ohio; Miss Dora Jones, St. Louis, Mo.; Dr. John C. Le Grand, Anniston, Ala.; Dr. C. F. Taylor, William B. Saunders, Philadelphia, Pa.; Miss Hackedorn, Toledo, Ohio; Dr. F. E. Stewart, Detroit, Mich.; J. MacDonald, Jr., Irving J. Benjamin, Dr. Ferdinand King, Dr. H. P. Fairchild, New York City; Dr. R. W. Lowe, Bridgeport, Conn.; Dr. W. C. Wile, Danbury, Conn.; Dr. H. M. Simmons, Dr. William B. Canfield, Baltimore, Md.; H. A. Mathie, Dr. A. H. Ohman-Dumesnil, Dr. I. N. Love, St. Louis, Mo.; Dr. Landon B. Edwards, Richmond, Va.; Dr. Hudson, Austin, Texas; Dr. William F. Bartlett, Philadelphia; Dr. T. D. Crothers, Hartford, Conn.; Dr. Gilbert I. Cullen, Cincinnati, Ohio; Dr. Henry S. Upson, Cleveland, Ohio; Dr. E. E. Holt, Portland, Maine; J. M. Grosvenor, Jr., Boston; Charles Wood Fassett, St. Joseph, Mo.

Nineteen new members were admitted and questions of the day affecting medical publishers were profitably discussed.

Beginning with July 1st, a monthly bulletin will be issued for the benefit of members of the Association. It is to be edited by Drs. P. H. Fairchild, J. MacDonald, Jr., and Ferdinand King, New York City; Dr. J. C. Le Grand, of Anniston, Ala.; and Charles Wood Fassett, of St. Joseph, Mo.

The Secretary was authorized to issue in pocket form a revised list of medical advertisers.

Upon invitation, the Association banqueted with the Medical Editors, on Monday evening.

The officers re-elected were as follows: President, Dr. Landon B. Edwards, of Richmond, Va.; Vice-President, Dr. H. C. Culbertson, Cincinnati, Ohio; Treasurer, J. MacDonald, Jr., New York City; Secretary, Charles Wood Fassett, St. Joseph, Mo. Dr. J. C. Le Grand and Irving J. Benjamin were elected on the Executive Board.

